# Equity trends in ownership of insecticide-treated nets in 19 sub-Saharan African countries

**DOI:** 10.2471/BLT.16.172924

**Published:** 2017-02-02

**Authors:** Cameron Taylor, Lia Florey, Yazoume Ye

**Affiliations:** a The DHS Program, ICF, 530 Gaither Road, Suite 500, Rockville, MD 20850, United States of America (USA).; b MEASURE Evaluation, ICF, Rockville, USA.

## Abstract

**Objective:**

To examine the change in equity of insecticide-treated net (ITN) ownership among 19 malaria-endemic countries in sub-Saharan Africa before and after the launch of the Cover The Bed Net Gap initiative.

**Methods:**

To assess change in equity in ownership of at least one ITN by households from different wealth quintiles, we used data from Demographic and Health Surveys and Malaria Indicator Surveys. We assigned surveys conducted before the launch (2003–2008) as baseline surveys and surveys conducted between 2009–2014 as endpoint surveys. We did country-level and pooled multicountry analyses. Pooled analyses based on malaria transmission risk, were done by dividing geographical zones into either low- and intermediate-risk or high-risk. To assess changes in equity, we calculated the Lorenz concentration curve and concentration index (C-index).

**Findings:**

Out of the 19 countries we assessed, 13 countries showed improved equity between baseline and endpoint surveys and two countries showed no changes. Four countries displayed worsened equity, two favouring the poorer households and two favouring the richer. The multicountry pooled analysis showed an improvement in equity (baseline survey C-index: 0.11; 95% confidence interval, CI: 0.10 to 0.11; and endpoint survey C-index: 0.00; 95% CI: −0.01 to 0.00). Similar trends were seen in both low- and intermediate-risk and high-risk zones.

**Conclusion:**

The mass ITN distribution campaigns to increase coverage, linked to the launch of the Cover The Bed Net Gap initiative, have led to improvement in coverage of ITN ownership across sub-Saharan Africa with significant reduction in inequity among wealth quintiles.

## Résumé

**Objectif:**

Examiner les changements au niveau de l'égalité de possession de moustiquaires imprégnées d'insecticide (MII) dans 19 pays d'Afrique subsaharienne où le paludisme est endémique, avant et après le lancement de l'initiative *Cover the bed net gap*.

**Méthodes:**

Afin d'évaluer les changements concernant l'égalité de possession d'au moins une MII par ménage, dans différents quintiles de richesse, nous avons utilisé des données provenant des enquêtes démographiques et sanitaires ainsi que des enquêtes sur les indicateurs du paludisme. Les enquêtes menées avant le lancement de l'initiative (2003–2008) ont été prises comme enquêtes de référence et celles menées de 2009 à 2014 comme enquêtes finales. Nous avons effectué des analyses par pays ainsi que des analyses regroupées multi-pays. Les analyses regroupées en fonction du risque de transmission du paludisme ont été effectuées en divisant les zones géographiques selon que le risque était faible ou intermédiaire ou bien élevé. Pour évaluer les changements en matière d'égalité, nous avons calculé la courbe de concentration de Lorenz ainsi que l'indice de concentration (indice C).

**Résultats:**

Sur les 19 pays que nous avons étudiés, 13 montraient une amélioration de l'égalité entre l'enquête de référence et l'enquête finale, et deux pays ne présentaient aucun changement. Quatre pays affichaient une moins bonne égalité: deux qui favorisaient les ménages les plus pauvres et deux les plus riches. L'analyse regroupée multi-pays a révélé une amélioration de l'égalité (indice C de l'enquête de référence: 0,11; intervalle de confiance (IC) de 95%: 0,10 à 0,11; indice C de l'enquête finale: 0,00; IC 95%: −0,01 à 0,00). Des tendances similaires ont été observées entre les zones à risque faible et intermédiaire et celles à risque élevé.

**Conclusion:**

Les campagnes de distribution massive de MII visant à améliorer la couverture, associées au lancement de l'initiative *Cover the bed net gap*, ont permis d'augmenter le taux de possession de MII en Afrique subsaharienne et de réduire significativement les inégalités entre quintiles de richesse.

## Resumen

**Objetivo:**

Examinar el cambio en la equidad de propiedad de mosquiteros tratados con insecticida (ITN) en 19 países donde la malaria es endémica en el África subsahariana, antes y después del lanzamiento de la iniciativa *Cover the bed net gap*.

**Métodos:**

Para evaluar el cambio en la equidad de propiedad de, al menos, un ITN por domicilio de distintos sectores demográficos, se utilizó información proveniente de las Encuestas de Demografía y Salud y las Encuestas de Indicadores de Malaria. Se asignaron las encuestas realizadas antes del lanzamiento (entre 2003 y 2008) como encuestas iniciales y las realizadas entre 2009 y 2014 como encuestas finales. Se realizaron análisis a nivel nacional y análisis conjuntos entre países. Los análisis conjuntos realizados en base al riesgo de transmisión de la malaria se llevaron a cabo dividiendo las zonas geográficas por riesgo bajo e intermedio o alto. A fin de evaluar los cambios en la equidad, se calcularon la curva de concentración de Lorenz y el índice de concentración (índice C).

**Resultados:**

De los 19 países evaluados, 13 mostraron una equidad mejorada entre las encuestas iniciales y las finales, y dos no mostraron cambios. Cuatro países mostraron una reducción de la equidad, dos favoreciendo a los domicilios más pobres y otros dos a los más ricos. El análisis conjunto realizado en varios países mostró una mejora en la equidad (índice C de la encuesta inicial: 0,11; intervalo de confianza, IC, del 95%: de un 0,10 a un 0,11; e índice C de la encuesta final: 0,00; IC del 95%: de un -0,01 a un 0,00). Se observaron tendencias similares tanto en las zonas de riesgo bajo e intermedio como en las zonas de riesgo alto.

**Conclusión:**

Las campañas de distribución en masa de ITN para aumentar la cobertura (vinculadas al lanzamiento de la iniciativa Cover the bed net gap) han conllevado una mejora en la cobertura de propiedad de ITN en toda el África subsahariana, con una reducción importante de la desigualdad entre los sectores demográficos.

## ملخص

**الغرض:**

فحص التغير في نسبة مساواة في امتلاك الناموسيات المُعالجة بمبيد حشري (ITN) بين 19 دولة من الدول التي تنتشر فيها الملاريا الواقعة بجنوب الصحراء الأفريقية قبل انطلاق مبادرة "تغطية السرير بالناموسية" وبعد انطلاقها.

**الطريقة:**

تقييم التغيير الطارئ على المساواة في امتلاك العائلات ضمن فئات خمسية سكانية مختلفة لناموسية واحدة معالجة بمبيد حشري على الأقل، واستخدمنا البيانات الواردة من الاستطلاعات المرتبطة بالخصائص السكانية والجوانب الصحية والمسوح المتعلقة بالملاريا. وقمنا بإسناد المسوح التي أجريناها قبل الانطلاق (2003 – 2008) كمسوح أساسية والمسوح التي أجريناها في الفترة ما بين 2009 – 2014 كمسوح لنقطة النهاية. وقمنا بإجراء تحاليل على المستوى القطري وأخرى تجمع بين عدة بلدان. وتم إجراء التحاليل الجامعة المعتمدة على خطر انتشار الملاريا بواسطة تقسيم المناطق السكنية إلى مناطق بنسب خطر منخفضة ومتوسطة، ومناطق أخرى بنسب خطر مرتفعة. لتقييم التغيرات الطارئة على المساواة، قمنا بحساب منحنى تركيز لورنز ومؤشر التركيز (C-index).

**النتائج:**

ضمن 19 دولة خضعت للتقييم، أظهرت 13 دولة تحسنًا في المساواة بين المسوح الأساسية ومسوح لنقطة النهاية، ولم تُظهِر دولتان أي تغييرات. وأظهرت أربع دول درجة رديئة من المساواة، حيث فضلت دولتان الأُسر الأفقر فيما فضلت دولتان الُأسر الأغنى. كما أظهر التحليل الجامع لعدة بلدان تحسنًا في المساواة (مؤشر تركيز للمسح الأساسي يبلغ: 0.11؛ ‏95% كمقدار لنسبة الأرجحية: 0.10 إلى 0.11، ومؤشر تركيز مسح نقطة النهاية: 0.00؛ ‏95‏% كمقدار لنسبة الأرجحية: -0.01 إلى 0.00). كما ظهرت اتجاهات متشابهة في المناطق ذات مستوى الخطر المنخفض والمتوسط ومستوى الخطر المرتفع.

**الاستنتاج:**

أدى الانتشار الهائل لحملات امتلاك الناموسيات المعالجة بمبيد حشري لزيادة التغطية – مع انطلاق مبادرة *تغطية السرير بالشبكة* – إلى حدوث تحسن في تغطية امتلاك الناموسية المعالجة بمبيد حشري عبر الدول الأفريقية الواقعة بجنوب الصحراء مع انخفاض ملحوظ في عدم المساواة بين الفئات السكانية الخمسية.

## 摘要

**目的:**

旨在调查 19 个撒哈拉以南非洲国家在推行*消除蚊帐差异*举措前后拥有杀虫剂浸泡蚊帐 (ITN) 的公平性变化。

**方法:**

为了评估不同财富五分位家庭至少拥有一个杀虫剂浸泡蚊帐 (ITN) 的公平性变化，我们使用了“人口与健康调查”和“疟疾检测指标调查”中的数据。我们将举措推行前（2003-2008 年）开展的调查指定为基线调查，而将 2009-2014 年间的调查指定为端点调查。 我们开展了国家层面上的分析和多国家汇总分析。汇总分析基于疟疾传播风险，通过将地理区域划分成中低风险或高风险来展开。为了评估公平性变化，我们计算了洛伦兹浓度曲线和浓度指标（C-指标）。

**结果:**

在我们评估的 19 个国家中，有 13 个国家在基线和端点调查之间表现出公平性有所提高，两个国家没有表现出任何变化。四个国家呈现出公平性降低，其中两个国家有利于更贫穷的家庭，另两个国家有利于更富裕的家庭。多国家汇总调查表现出公平性有所提高（基线调查 C-指标： 0.11；95% 置信区间，CI： 0.10 至 0.11；端点调查 C-指标： 0.00; 95% CI: 0.01 至 0.00）。 中低风险和高风险区的趋势与之类似。

**结论:**

为提高覆盖率而进行的大规模杀虫剂浸泡蚊帐 (ITN) 与*消除蚊帐差异*举措的推行相呼应，提高了撒哈拉以南非洲国家杀虫剂浸泡蚊帐 (ITN) 的覆盖率，并且显著降低了财富五分位家庭间的不公平性。

## Резюме

**Цель:**

Изучить изменения в степени равномерности распределения обработанных инсектицидами противомоскитных сеток в 19 эндемичных по малярии странах Африки к югу от Сахары до и после запуска инициативы *Преодоление неравенства в распределении надкроватных сеток*.

**Методы:**

Чтобы оценить изменение степени равномерности распределения по крайней мере одной обработанной инсектицидами противомоскитной сетки на семью из разных квинтилей доходов, авторы использовали данные демографических обследований и обследований состояния здоровья населения, а также данные обследований по показателям малярии. Обследования, проведенные до запуска инициативы (2003–2008 гг.), были определены как первоначальные обследования, а обследования, проведенные в 2009–2014 гг., — как заключительные. Были проведены анализы на уровне страны и объединенные анализы по нескольким странам. Объединенные анализы, основанные на риске заражения малярией, были проведены путем разделения географических зон на зоны с низким, средним и высоким уровнем риска. Для оценки изменения степени равномерности авторы рассчитали кривую концентрации Лоренца и показатель концентрации (C-индекс).

**Результаты:**

Из 19 стран, подвергнутых оценке, в 13 наблюдалось увеличение степени равномерности в период между первоначальными и заключительными обследованиями, а в двух странах изменения отсутствовали. В четырех странах степень равномерности уменьшилась, причем в двух из них сетки были более распространены среди более бедных семей, а в двух других — среди более богатых. Согласно результатам объединенного анализа по нескольким странам, степень равномерности увеличилась (C-индекс при первоначальных обследованиях: 0,11; 95%-й доверительный интервал, ДИ: 0,10–0,11; C-индекс при заключительных обследованиях: 0,00; 95%-й ДИ: −0,01–0,00). Аналогичные тенденции наблюдались в зонах с низким, средним и высоким риском.

**Вывод:**

Кампании по массовому распространению обработанных инсектицидами противомоскитных сеток, связанные с запуском инициативы *Преодоление неравенства в распределении надкроватных сеток*, привели к увеличению охвата обработанными инсектицидами противомоскитными сетками в разных странах Африки к югу от Сахары на фоне значительного сокращения неравенства среди квинтилей дохода.

## Introduction

Equity in health is a major tenet of global development organizations, such as the World Health Organization (WHO) and the World Bank, whose policies are aimed to decrease the gap between poor and rich populations. WHO defines health inequity as “inequality with respect to health determinants, access to the resources needed to improve and maintain health or health outcomes”.^1^ Many diseases, such as malaria, are not distributed equitably among populations. Malaria disproportionately affects poor, rural populations, with pregnant women and young children at highest risk of severe morbidity and mortality.^2^^–^^10^ Addressing inequities that are actionable, such as the availability of commodities, has been the cornerstone of malaria control efforts for more than a decade.

In April 2008 the Roll Back Malaria Partnership, together with the Secretary-General of the United Nations, launched the initiative Cover The Bed Net Gap to achieve the goal of universal bed-net coverage by December 2010.^11^^,^^12^ The aim of this initiative is to have every household at risk of malaria transmission and every person within that household protected by an insecticide-treated net (ITN).^13^^,^^14^ Since the launch of the initiative, countries have achieved high ITN coverage levels using various distribution channels such as community delivery, routine health services or outreach activities.

Before the launch of the initiative, many distribution strategies focused on populations at higher risk of malaria. The ITN policies frequently included distribution of nets to caregivers of children younger than five years during routine vaccination campaigns, distribution to pregnant women during antenatal care visits and via social marketing. In addition, ITNs could be purchased either at health facilities or in the private market. These distribution strategies led to inequity in ITN ownership among subgroups,^2^^,^^15^ particularly between socioeconomic subgroups. Richer households were more likely to own ITNs than the poorest households, probably as a result of low access to health care among the poorest populations.^2^^,^^16^^,^^17^ With the launch of the initiative, the distribution of ITNs shifted from targeted distribution to mass distribution campaigns. This shift gave malaria control programmes the opportunity to reduce disparities among subgroups by increasing ITN ownership^18^^–^^24^ to reduce the malaria burden.^25^

The mass distribution campaigns aim to provide one ITN for every two household members. Based on the longevity of the nets and the cost–effectiveness of conducting a mass distribution as compared to a targeted net replacement, these campaigns are recommended to take place every three years.^26^

In 2015, seven years after the launch of the initiative, few multicountry studies have documented the effect of the mass distribution strategy on equity in ITN ownership coverage in sub-Saharan Africa. This study assesses the level of equity in bed-net ownership before and after the widespread implementation of national ITN distribution strategies.

## Methods

### Data

We used data from Demographic and Health Surveys (DHS) and Malaria Indicator Surveys (MIS), which are nationally representative, population-based household surveys and which use validated standard methods in all countries. Further details can be found elsewhere.^27^ The analysis focused on malaria-endemic countries in sub-Saharan Africa that have conducted DHS or MIS between 2003 and 2014. We defined surveys made before the launch of the initiative as baseline surveys and these were conducted between the years 2003–2008. Surveys carried out between years 2009–2014, we defined as endpoint surveys. All surveys are independent from each other.

### Analysis

We did two sets of analyses: a country-level equity trend analysis of ITN ownership and a multicountry pooled analysis examining equity of ITN ownership by malaria transmission risk zones. Inclusion criteria for the country-level equity trend analyses included: (i) countries with one survey conducted between years 2003–2008 (baseline) and the other survey conducted between years 2009–2014 (endpoint); and (ii) all surveys must have included data on ITN ownership via a bed-net roster in the household questionnaire.

To explore if equity in ITN ownership varies by level of malaria transmission, the multicountry pooled equity analysis categorized all survey clusters into categories of malaria risk. We included surveys that had publicly available global positioning system (GPS) data for the surveyed clusters. If a country had more than one survey with GPS data in a time period, we used the most recent survey in both the baseline and endpoint. However, in Rwanda, we used the 2010 DHS GPS coordinates for the endpoint due to a lack of GPS coordinates for the most recent 2013 MIS survey. Only countries with GPS data from both surveys were included in the pooled analysis.

#### Defining ITN ownership

The outcome of interest is household ITN ownership, defined as the proportion of households with at least one ITN. As recommended by the Roll Back Malaria Monitoring and Evaluation Reference Group, the indicator is standard across countries and reflects the extent to which ITN distribution campaigns have reached all households.^28^ For each survey, we calculated the proportion of households with at least one ITN. To test for significant changes in ITN ownership between baseline and endpoint surveys, we calculated 95% confidence intervals (CI).

We did not use the indicator for universal bed-net coverage, i.e. the proportion of households with at least one ITN for every two people, since the indicator was not launched until 2008 and therefore not captured in baseline surveys.

#### Defining wealth quintiles

The DHS wealth index measures economic well-being of households independently from health and education.^29^^,^^30^ The DHS wealth index is a survey-specific measure of the relative economic status of households based on analysis of household assets and service amenities at a particular point in time. Wealth quintiles (lowest, second, middle, fourth, and highest) ranking indicates relative rather than absolute economic status of the household.^30^^–^^32^

#### Defining malaria endemicity

We assigned each household cluster into geographical zones based on malaria transmission risk. To link DHS and MIS geo-coordinates (latitude, longitude) of each survey cluster to transmission risk zones, we used geo-coordinated *Plasmodium falciparum* parasite prevalence rates among children aged 2–10 years (*Pf*PR_2–10_) from the Malaria Atlas Project 2010.^33^ We assigned all households in a cluster from the DHS or MIS survey data to the same malaria transmission risk zone based on corresponding *Pf*PR_2–10_ data for that cluster.^34^ For the transmission zone categories, we used the standard *Pf*PR_2–10_ cut-offs from the Malaria Atlas Project: no-risk: *Pf*PR_2–10_ < 0.1%; low-risk: 0.1% > *Pf*PR_2–10_ ≤ 5%; intermediate-risk: 5% > *Pf*PR_2–10_ ≤ 40%; and high-risk: *Pf*PR_2–10_ > 40%.^35^

Out of the 346 272 household clusters located in 15 countries, 50% (173 136) were categorized in the high-risk category, 36% (124 658) in the intermediate-risk category, 10% (34 627) in the low-risk category and 4% (13 851) in the no-risk category. We excluded clusters located in areas with no risk of malaria from analyses because populations in these areas would not be targeted by ITN distribution campaigns. Due to small sample size in the low-risk group, we combined the intermediate and the low-risk groups.

#### Equity calculation

We used the Lorenz concentration curve (C-curve) and the Lorenz concentration index (C-index) to assess equity in household ITN ownership across household wealth quintiles. The C-curve graphically presents the degree of economic-related inequality.^36^^,^^37^ In the C-curve graphs, the *x*-axis presents the cumulative percentage of the sample, ranked by wealth, beginning with the poorest, while the *y*-axis presents the cumulative percentage of the variable of interest corresponding to the cumulative percentage of the distribution of wealth.^36^ In the C-curve graphs, the dashed 45° line represents equity whereby the health outcome is distributed equally among all wealth quintiles. The C-curve will be below the equity line if ITN ownership is concentrated in richer households and will be above the equity line if ITN ownership is predominantly among poorer households.

The C-index, which provides quantification of this measure of equity, is defined as twice the area between the C-curve and the 45° line of equity. We calculated the C-index values by using the following equation *C* = (*P_1_L_2_−P_2_L_1_*)*+*(*P_2_L_3_−P_3_L_2_*)*+…+*(*P_t-1_L_t_−P_t_L_t-1_*), where *P* is the cumulative percentage of the household ranked by economic status in group *t*, and *L* is the corresponding concentration curve ordinate.^37^ C-index values range between −1 to 1. A value of 0 suggests no difference in ITN ownership between different wealth quintiles. A C-index larger than 0 suggests that ITN ownership is predominantly among the richer households. Conversely, a negative index indicates that ITN ownership is more concentrated among the poorer households.^17^^,^^38^

We used the *concindc* command in Stata version 13 (StataCorp. LP, College Station, United States of America) to calculate the C-index values and their standard errors and we used the *clorenz* command for producing the C-curves. We calculated 95% CI for the C-index values.

## Results

In total, 19 countries (45 surveys) met the inclusion criteria for country-level equity analysis. For the multicountry pooled equity analysis, we included 15 countries (30 surveys; Table 1).

**Table 1:** Countries included in country-level and pooled equity analysis for insecticide-treated net ownership, sub-Saharan Africa, 2003–2014

Country	Baseline survey, type and year	Mid-point survey, type and year	Endpoint survey, type and year	*Pf*PR_2–10_,^a^ %	Included in pooled analysis^b^
Angola	2006–2007 MIS	N/A	2011 MIS	8.1	Yes
Benin	2006 DHS	N/A	2011–12 DHS	29.9	No
Burkina Faso	2003 DHS	N/A	2010 DHS	65.4	Yes
Cameroon	2004 DHS	N/A	2011 DHS/MICS	23.5	Yes
Congo	2005 DHS	N/A	2011–2012 DHS	17.5	No
Democratic Republic of the Congo	2007 DHS	N/A	2013–2014 DHS	48.0	Yes
Guinea	2005 DHS	N/A	2012 DHS/MICS	42.4	Yes
Madagascar	2008–2009 DHS	2011 MIS	2013 MIS	5.8	Yes
Malawi	2004 DHS	2010 DHS	2012 MIS	35.6	Yes
Mali	2006 DHS	N/A	2012–2013 DHS	32.0	Yes
Mozambique	2007 MIS	N/A	2011 DHS	35.5	No
Niger	2006 DHS	N/A	2012 DHS	29.3	No
Nigeria	2008 DHS	2010 MIS	2013 DHS	32.5	Yes
Rwanda	2005 DHS	2010 DHS	2013 MIS	2.3	Yes
Senegal	2005 DHS	2008–2009 MIS	2010–2011 DHS	5.8	Yes
Sierra Leone	2008 DHS	N/A	2013 DHS	52.8	Yes
Uganda	2006 DHS	2009 MIS	2011 DHS	37.0	Yes
United Republic of Tanzania	2004–2005 DHS	2007–2008 THMIS	2011–2012 THMIS	10.6	Yes
Zimbabwe	2005–2006 DHS	N/A	2010–2011 DHS	2.5	Yes

DHS: Demographic Health Survey; MICS: Multiple Indicator Cluster Surveys; MIS: Malaria Indicator Survey; N/A: not available; P*f*PR_2–10_: *Plasmodium falciparum* parasite rate; THMIS: Tanzania human immunodeficiency virus and malaria indicator survey.

^a^ PfPR_2–10_ is the national mean of *Plasmodium falciparum* parasite rate in children aged 2–10 years and was obtained from the 2010 Malaria Atlas Project.^33^

^b^ Countries with no GPS data for both surveys were excluded from the pooled-analysis.

### ITN ownership

**Fig. 1:**
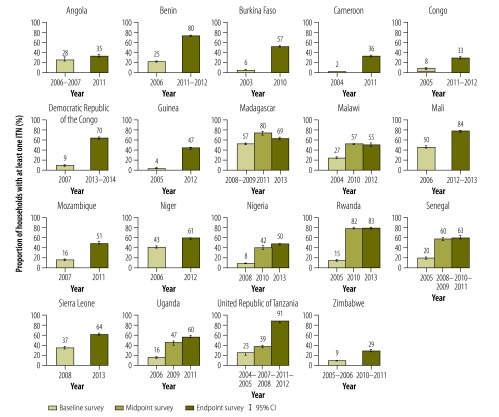
Proportion of households with at least one insecticide-treated net by country and survey year, 19 countries in sub-Saharan Africa, 2003–2014

CI: confidence interval; ITN: insecticide-treated net.

In all countries, except Angola, there was a statistically significant increase in ITN ownership between the baseline and endpoint surveys. Rwanda and the United Republic of Tanzania showed the greatest improvement in ITN ownership, from 15% to 83% and from 23% to 91%, respectively. Angola displayed the smallest improvements in ITN ownership (from 28% to 35%; Fig. 1).

### Country-level equity analysis

At the country level, 13 out of the 19 countries showed improvements in equity of ITN ownership between baseline and endpoint surveys, while two countries showed no changes and four countries displayed worsened equity (Table 2).

**Table 2:** Equity changes in ownership of insecticide-treated nets before and after the launch of the Cover The Bed Net Gap initiative, sub-Saharan Africa, 2003–2014

Equity change	Country	C-index (95% CI)	
		Baseline survey	Endpoint survey
**Improved**			
From ownership concentrated in richer households to more equitable ownership, but still concentrated in richer households	Burkina Faso	0.45 (0.40 to 0.49)	0.06 (0.05 to 0.06)
	Democratic Republic of the Congo	0.26 (0.23 to 0.30)	0.03 (0.03 to 0.04)
	Malawi	0.29 (0.28 to 0.31)	0.06 (0.04 to 0.07)
	Rwanda	0.35 (0.33 to 0.38)	0.02 (0.02 to 0.03)
	Uganda	0.11 (0.09 to 0.14)	0.02 (0.02 to 0.03)
From ownership concentrated in richer households to equitable ownership	Benin	0.23 (0.21 to 0.24)	0.00 (−0.01 to 0.00)
	Cameroon	0.25 (0.17 to 0.33)	0.02 (0.00 to 0.03)
	United Republic of Tanzania	0.41 (0.39 to 0.43)	−0.01 (−0.01 to 0.00)
From ownership concentrated in richer households to more equitable ownership but concentrated in poorer households	Congo	0.15 (0.10 to 0.20)	−0.11 (−0.12 to −0.09)
	Guinea	0.28 (0.21 to 0.35)	−0.03 (−0.04 to −0.02)
	Nigeria	0.18 (0.16 to 0.20)	−0.06 (−0.06 to −0.05)
	Sierra Leone	0.05 (0.04 to 0.07)	−0.02 (−0.03 to −0.01)
	Zimbabwe	0.19 (0.15 to 0.22)	−0.06 (−0.08 to −0.04)
**No change**			
Ownership equitably distributed in both surveys	Mali	0.02 (0.00 to 0.03)	0.00 (0.00 to 0.01)
Ownership was concentrated in richer households in both surveys	Mozambique	0.05 (0.02 to 0.08)	0.04 (0.03 to 0.05)
**Worsened**			
Favouring poorer households			
From ownership concentrated in poorer households to increased ownership concentrated in poorer households	Madagascar	−0.04 (−0.04 to −0.03)	−0.06 (−0.07 to −0.05)
	Senegal	−0.01 (−0.05 to 0.00)	−0.11 (−0.12 to −0.10)
Favouring richer households			
From equitable ownership or ownership concentrated in richer households to concentrated ownership in richer households	Angola	0.05 (0.01 to 0.08)	0.17 (0.15 to 0.18)
	Niger	0.00 (−0.02 to 0.01)	0.09 (0.08 to 0.10)

CI: confidence interval; C-index: concentration index; ITN: insecticide-treated net.

Notes: Cover The Bed Net Gap was an initiative launched by the Roll Back Malaria Partnership in April 2008.^12^ Years of the surveys are given in Table 1. A value of 0 suggests no difference in ITN ownership between different wealth quintiles. A C-index larger than 0 suggests that ITN ownership is concentrated among the richer households. Conversely, a negative index indicates that ITN ownership is more concentrated among the poorer households.^17^^,^^38^

**Fig. 2:**
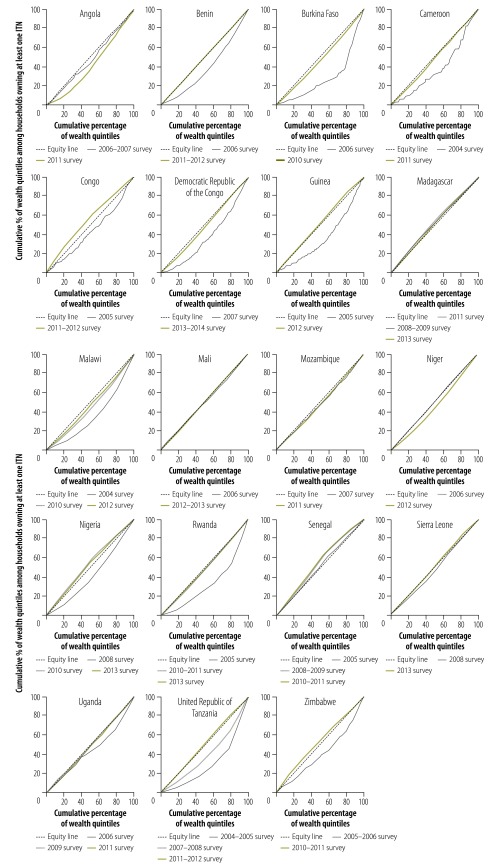
Equity changes in insecticide-treated net ownership by country, 19 countries in sub-Saharan Africa, 2003–2014

For all countries showing improvements in equity, the ITN ownership was concentrated in households from the highest wealth quintiles in the baseline surveys, as indicated by a C-curve below the equity line (C-index > 0). However, the countries showed different levels of improvement. For Burkina Faso, the Democratic Republic of the Congo, Malawi, Rwanda and Uganda equity significantly improved with C-index values closer to zero in the endpoint surveys. For Benin, Cameroon and the United Republic of Tanzania, equity in ITN ownership across wealth quintiles (C-index = 0) had been achieved. The endpoint surveys from the Congo, Guinea, Nigeria, Sierra Leone and Zimbabwe showed higher levels of ITN ownership among poorer households (C-curve above the equity line and C-index < 0; Fig. 2; Table 2).

ITN: insecticide-treated net.

Note: A survey line under the equity line denotes that ITN ownership was more concentrated in richer households, while a survey line above the equity line represents ownership favouring the poorer households.

In Mali, ITN ownership was equally distributed across wealth quintiles in both baseline and endpoint surveys with no significant change. In Mozambique, ITN ownership remained concentrated among richer households in the endpoint survey. However, C-index values were close to zero (Fig. 2; Table 2).

Madagascar and Senegal maintained levels of inequity that favoured the poorest households. In Angola and Niger, while the inequity in the baseline surveys was close to zero, in the endpoint surveys, household ITN ownership increased and was in favour of the richer households (Fig. 2; Table 2).

**Fig. 3:**
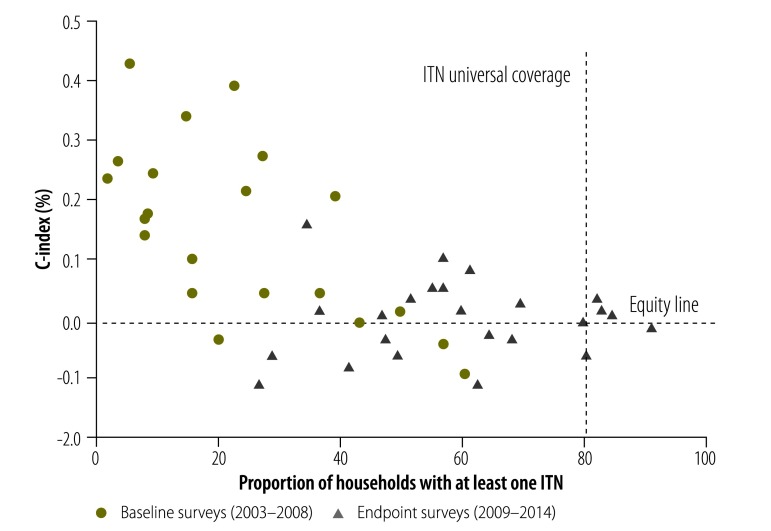
Proportion of households with at least one insecticide-treated net, by concentration index, sub-Saharan Africa, 2003–2014

Fig. 3 shows a scatter plot of the C-index by ITN ownership for all surveys included in the country-level analyses. The plot indicates a decline in the disparity of the C-index values as ITN coverage increases. Surveys that took place between 2009–2014 have higher levels of ITN ownership and greater equity compared to surveys from 2003–2008.

C-index: concentration-index; ITN: insecticide-treated net.

Note: We used the Lorenz C-index to assess equity in household ITN ownership across household wealth quintiles. ITN universal coverage of 80% is based on the Global Strategic Plan 2005–2015.^39^

### Pooled equity analysis

#### All countries

**Fig. 4:**
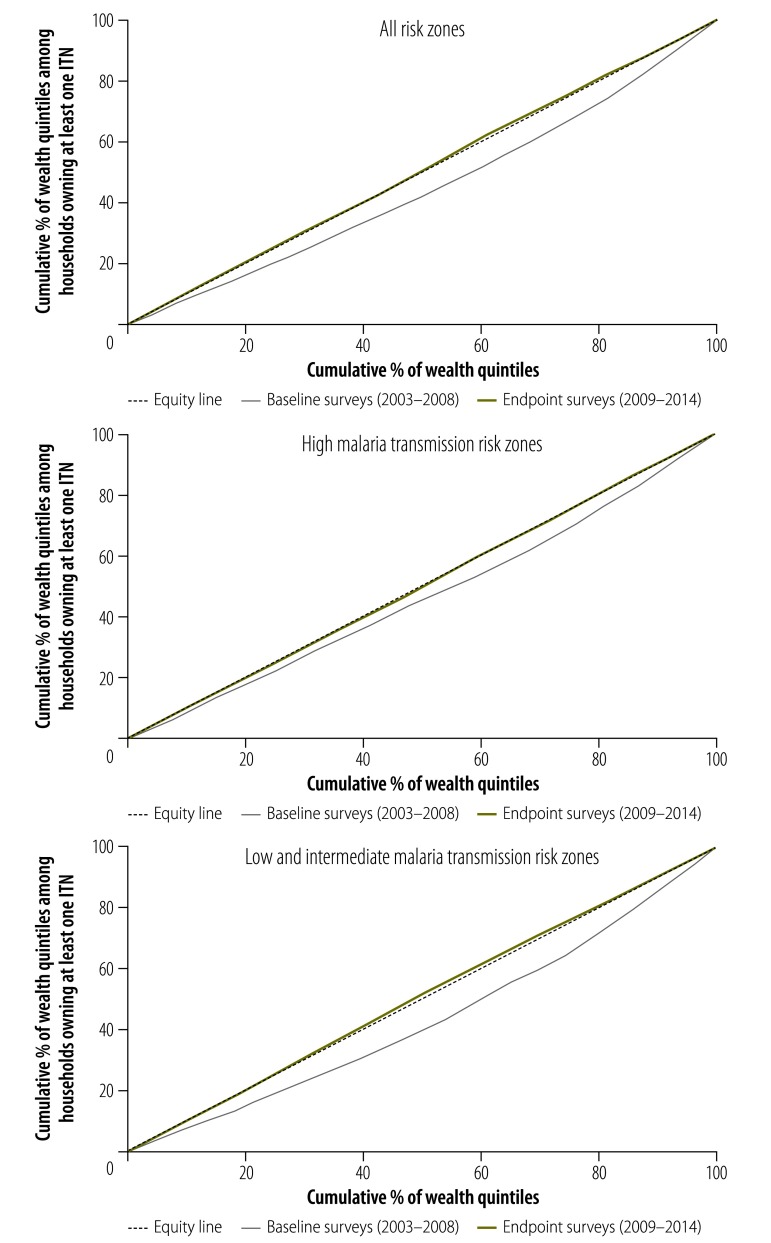
Equity changes in insecticide-treated net ownership by malaria transmission risk zone, 15 countries in sub-Saharan Africa, 2003–2014

The multicountry pooled analysis indicates a significant improvement in ITN ownership equity between baseline (C-index: 0.11; 95% CI: 0.10 to 0.11) and endpoint surveys (C-index: 0.00; 95% CI: −0.01 to 0.00; Fig. 4)

ITN: insecticide-treated net.

Notes: Each household cluster was assigned a malaria transmission risk category based on *Plasmodium falciparum* parasite prevalence rates among children aged 2–10 years (*PfPR*_2–10_) from the Malaria Atlas Project 2010.^33^ We used the standard *PfPR*_2–10_ cut-offs from the Malaria Atlas Project: low and intermediate risk: 0.1% >  *PfPR*_2–10_ < 40% and high risk: *PfPR*_2–10_ > 40%. A survey line under the equity line denotes that ITN ownership was more concentrated in richer households, while a survey line above the equity line represents ownership favouring the poorer households.

#### By transmission risk

In high malaria transmission risk zones, ITN ownership was concentrated in households from the higher wealth quintiles in the baseline surveys (C-index: 0.07; 95% CI: 0.06 to 0.08). However, in the endpoint surveys this inequity was no longer evident (C-index: 0.00; 95% CI: 0.00 to 0.01; Fig. 4). In the low and intermediate malaria transmission risk zones, the ITN ownership was in favour of richer households in the baseline surveys (C-index: 0.14; 95% CI: 0.13 to 0.14), but shifted to favour the poorer households in the endpoint surveys (C-index: −0.01, 95% CI: −0.02 to −0.01; Fig. 4).

## Discussion

This study presents evidence of the positive impact of mass ITN distribution strategies on equity of ITN ownership in 19 malaria-endemic countries in sub-Saharan Africa. In 15 of the 19 countries analysed, ITN ownership either became more equitable or maintained equity between baseline and endpoint surveys. In four countries, improvements in equity could not be detected between the two surveys: in two countries the ITN ownership remained concentrated among households from the lowest wealth quintiles, while in the other two, ITN ownership remained concentrated among the richer households.

The pooled multicountry analyses further supported the findings that the significant increase in ITN ownership has favoured the poorest households in most settings. In the countries where ITN ownership either became more equitable or maintained equity, all showed a significant increase in the proportion of households with at least one ITN between baseline and endpoint surveys. In countries with very high levels of household ITN ownership, such as Rwanda and the United Republic of Tanzania, the chances of equitable distribution are inherently higher. Thus, the recent funding for malaria control^25^ and the subsequent investment in mass ITN distribution campaigns have likely contributed to reduced inequity among wealth quintiles by increasing overall coverage.

Angola, Mali, Mozambique and Niger had close to equitable ITN ownership in their baseline surveys despite relatively low overall levels of ITN ownership. This finding could be due to early implementation of focused nationwide campaigns where ITN distributions were integrated with child health campaigns.^19^^,^^40^^–^^42^ In Mali and Mozambique, ITN ownership remained equitably distributed in endpoint surveys, possibly due to the rollout of net-distribution campaigns to achieve universal coverage in 2008 and 2011.^43^^,^^44^ However, Angola and Niger experienced a decrease in equity in their endpoint surveys despite low inequity at baseline. The trend of increasing inequity in Angola could be partially due to the timing of campaigns in relationship to the survey as well as a shift in ITN distribution from integrated campaigns to distribution in only selected municipalities.^45^ Reasons for the worsened equity in Niger is less clear, but possible explanations could include the lack of implementation of free ITN distribution campaigns between baseline and endpoint surveys.^19^^,^^25^ Madagascar and Senegal were the only countries that maintained levels of inequity from baseline to endpoint in favour of households from the lowest wealth quintiles.

The trend seen in Congo, Guinea, Nigeria, Sierra Leone and Zimbabwe – i.e. ITN ownership shifted from being concentrated in the richer households to being concentrated in the poorer households – is not surprising. These countries have moved towards universal ITN coverage for populations at-risk and shifted their distribution of ITNs to high-risk rural-areas, which are usually less wealthy than urban centres.^2^ Another reason for this trend may be that wealthier households have access to a wider range of other effective interventions, such as improved housing with screened windows and doors and closed eaves that make ITNs less essential for malaria prevention.

To explore if equity of ITN ownership varies by malaria transmission risk, we pooled clusters into two groups stratified by low/intermediate and high levels of malaria transmission. In the pooled analysis, equity increased significantly in both groups. However, the greatest improvement in equity occurred in clusters in low- and intermediate-risk zones. The observed results could be due to changing ITN policies between baseline and endpoint surveys, and more specifically, the rollout of free mass distribution campaigns after 2008. Before 2008, financial and logistic constraints caused most distribution campaigns to be targeted to high-risk populations (children younger than five years and pregnant women) and/or high-risk regions (rural, high-transmission zones). Therefore, households from the lowest wealth quintiles in low- and intermediate-risk zones were less likely to own a net if they did not have access to health-care services or could not afford to pay for a net at market price. The shift to free mass distribution campaigns may have improved equity by providing access to the households from the lowest wealth quintiles that did not have previous access to nets.

This study has a few limitations that should be highlighted. The wealth quintiles are based on assets, which may be different from country to country as the individual assets might have different weights in the principal component analysis. While the analysis used data from 19 countries, other countries may have experienced changes in equity, but were not captured in this analysis. In addition, we excluded four of the countries used in the country-level analysis from the pooled analysis due to a lack of GPS data. This study focused on equity of ITN ownership and did not assess ITN use or ITN access, i.e. the proportion of the population who could use an ITN with the assumption that one ITN can protect two individuals. Future studies should examine equity of ITN access as it is a more comprehensive measure of the level of protection within a household. With more countries implementing universal bed-net coverage strategies, capturing changes in equity of ITN access through survey data will be possible.

In conclusion, our findings support the hypothesis that national ITN distribution campaigns have increased ITN coverage and reduced economic inequity in ITN ownership since the launch of the Cover The Bed Net Gap initiative in 2008.^12^ However, further improvements are still needed to reach and maintain coverage targets. With the combination of increased ITN distribution through multiple adapted distribution mechanisms and monitoring inequities to ensure that the poorest are also get protected, great strides can be made towards malaria prevention across sub-Saharan countries.
